# Probabilistic Distances Between Trees

**DOI:** 10.1093/sysbio/syx080

**Published:** 2017-10-04

**Authors:** Maryam K Garba, Tom M W Nye, Richard J Boys

**Affiliations:** 1 *School of Mathematics & Statistics, Newcastle University, Newcastle upon Tyne NE1 7RU, UK*; 2 *Department of Mathematical Sciences, Bayero University, Kano, Nigeria*

## Abstract

Most existing measures of distance between phylogenetic trees are based on the geometry or topology of the trees. Instead, we consider distance measures which are based on the underlying probability distributions on genetic sequence data induced by trees. Monte Carlo schemes are necessary to calculate these distances approximately, and we describe efficient sampling procedures. Key features of the distances are the ability to include substitution model parameters and to handle trees with different taxon sets in a principled way. We demonstrate some of the properties of these new distance measures and compare them to existing distances, in particular by applying multidimensional scaling to data sets previously reported as containing phylogenetic islands. [Metric; probability distribution; multidimensional scaling; information geometry.

Many methods for postprocessing phylogenetic trees rely on some measure of distance between pairs of trees. A variety of different distances are used, for example: the Robinson–Foulds metric (Robinson and Foulds 1979, 1981), quartet distance (Estabrook et al. 1985), path-length-difference metric (Penny et al. 1993), Billera–Holmes–Vogtmann (BHV) metric (Billera et al. 2001; Owen and Provan 2011), and matching distance (Lin et al. 2012), among others. These are generally defined by directly comparing the branching pattern (usually called topology) and/or edge lengths in a given pair of trees. For example, the Robinson–Foulds metric is defined by counting the number of splits present in exactly one of a given pair of trees, while the BHV metric is defined in terms of splits and edge lengths. However, phylogenetic trees also represent probability models for genetic sequence data, and for some applications it might be more appropriate to use a distance measure which compares the probability distributions on characters induced by trees, rather than comparing the trees as geometric objects. This article describes methodology and software for calculating such distances and explores their properties. The software is available from http://www.mas.ncl.ac.uk/~ntmwn/probdist.

The following simple example illustrates the difference between the two approaches. Suppose we have two trees $T_1$ and $T_2$ with a common leaf-set such that the two trees differ only with respect to the position of a single taxon $x$. In other words, the subtrees of $T_1$ and $T_2$ obtained by removing $x$ are identical. Then, in the limit that the edge leading to $x$ gets increasingly long, distance measures which compare the topology or geometry of the trees will generally view $T_1$ and $T_2$ as being bounded away from each other (or getting further apart). However, under the same limit, the genetic sequence of $x$ effectively becomes independent of the other taxa. Since the relationships between the other taxa are fixed, the probability distributions on characters induced by $T_1$ and $T_2$ become identical in the limit, and so the distance tends to zero.

This article describes simulation methods which calculate (approximately) the Hellinger distance, Jensen–Shannon distance, and Kullback–Leibler divergence between trees when they are regarded as sequence models. These distance measures also have a natural extension to situations when trees do not share the same set of taxa. Unlike existing distance measures, the distance measures we propose can be defined between pairs $(T_i,{\mathbf{\theta}}_i)$, $i=1,2$, where $T_i$ is a tree and ${\mathbf{\theta}}_i$ is a vector of DNA substitution model parameters, rather than between trees alone. Phylogenetic trees are usually inferred with an associated substitution model, and so information is lost if comparison is only carried out on inferred trees without the associated substitution models.

The idea of identifying trees with points in a space of distributions on characters was first considered by Kim (2000). The space is usually referred to as the space of “hyperdimensional oranges” or “phylogenetic oranges.” Topological and combinatorial aspects of the space were studied by Moulton and Steel (2004). The methods developed in this article enable the computation of metrics on this space, a first step towards more involved geometrical methods such as computation of sample means and variances.

## Methods

### Probabilistic Distances

A binary character is an assignment of $\{0,1\}$ to each taxon, so there are $2^n$ characters in total for a tree with $n$ leaves. Likewise, a DNA character is an assignment of $\{A,C,G,T\}$ to each taxon, giving $4^n$ characters in total. We let $\Omega$ denote the set of states ($\{0,1\}$ or $\{A,C,G,T\}$) and use $\Omega^n$ to denote the set of all characters. Evolution over a tree is typically modeled by a Markov substitution process on the edges of the tree (Semple and Steel 2003). Any Markov process substitution model induces a distribution on characters at the leaves, that is, a distribution on $\Omega^n$. When $\Omega=\{0,1\}$, we assume the Markov substitution process is the unique symmetric process on two states. This Markov process has no parameters. When $\Omega=\{A,C,G,T\}$, we assume a general time-reversible (GTR) model with across-site Gamma rate heterogeneity. The parameters ${\mathbf{\theta}}$ for this model determine the rates of character substitution and the stationary distribution of the process. However, the methodology we develop below can be applied to arbitrary alphabets and substitution models, in particular, to amino acid models.

We look at distances between probability distributions on characters induced by the tree and substitution model; that is, look at distances 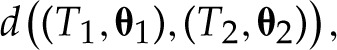 for trees $T_i$ and substitution model parameters ${\mathbf{\theta}}_{i}$. (Note that ${\mathbf{\theta}}_1$ and ${\mathbf{\theta}}_2$ are empty when the substitution process is the symmetric two-state model). The distance $d$ is either (i) the Hellinger (H) distance, (ii) Kullback–Leibler (KL) divergence, or (iii) Jensen–Shannon (JS) distance, and these are defined below.

Let $p({\mathbf{s}})$ and $q({\mathbf{s}})$, where ${\mathbf{s}}$ ranges over the set of characters, be probability mass functions for the probability distributions $p$ and $q$ on characters associated with two trees $T_1,T_2$ with common leaf-set of size $n$. The Hellinger distance between $p$ and $q$ is defined by

\begin{equation*}
d_H(p;q)^2 = \frac{1}{2} \sum_{{\mathbf{s}} \in \Omega^n} \left(\sqrt{p({\mathbf{s}})}-\sqrt{q({\mathbf{s}})}\right)^2.
\end{equation*}

The Hellinger distance $d_H$ is a metric bounded above by 1 (Gibbs and Su 2002). The Kullback–Leibler divergence of $p$ from $q$ is defined as

\begin{equation*}
d_{KL}(p;q) = \sum_{{\mathbf{s}}\in \Omega^n}p({\mathbf{s}})\log{\left(\frac{p({\mathbf{s}})}{q({\mathbf{s}})}\right)},
\end{equation*}

where log denotes the natural logarithm. The Kullback–Leibler divergence is not a metric because it is not symmetric and does not satisfy the triangle inequality. It is always non-negative (Gibbs and Su 2002). Finally, the JS distance is defined via the Kullback–Leibler divergence as

(1) \begin{equation*}\label{equ:JS} d_{JS}^{2}(p;q) = \frac{1}{2}d_{KL}\left(p;\frac{p+q}{2}\right)+\frac{1}{2}d_{KL}\left(q;\frac{p+q}{2}\right). \end{equation*}

It is a metric with an upper bound of $\sqrt{\log 2}$.

Computing these distance measures exactly for large trees is computationally expensive as there are $|\Omega|^n$ possible values for ${\mathbf{s}}$. However, we can estimate these distances via simulation since each can be expressed in terms of expectations with respect to the distributions $p$ and $q$ on characters. Suppose that ${\mathbf{s}}_{p,i}$, $i=1,\ldots,m$ are a set of $m$ characters simulated on tree $T_1$, and ${\mathbf{s}}_{q,i}$, $i=1,\ldots,m$ are a set of $m$ characters simulated on tree $T_2$. In other words, the characters ${\mathbf{s}}_{p,i}$ are independent samples from distribution $p$ and similarly for the characters ${\mathbf{s}}_{q,i}$. We can think these samples as each being equivalent to a simulated alignment with $m$ independent columns from the trees $T_1,T_2$, respectively. Then it can be shown that the Hellinger distance can be estimated via

\begin{equation*}
d_H(p;q)^{2} \simeq 1-\frac{1}{2m}\sum_{i=1}^{m}\left(\sqrt{\frac{q({\mathbf{s}}_{p,i})}{p({\mathbf{s}}_{p,i})}}+\sqrt{\frac{p({\mathbf{s}}_{q,i})}{q({\mathbf{s}}_{q,i})}}\right).
\end{equation*}

A full derivation is given in the Supplementary Appendix available on Dryad at http://dx.doi.org/10.5061/dryad.69bb2. Similarly, the Kullback–Leibler divergence can be estimated using

\begin{equation*}
d_{KL}(p;q) \simeq\frac{1}{m}\sum_{i=1}^{m}\log{\left(\frac{p({\mathbf{s}}_{p,i})}{q({\mathbf{s}}_{p,i})}\right)}
\end{equation*}

and this expression can be used to estimate the Jensen–Shannon metric using Equation (1).

Fixing a choice of distance measure between distributions, and given pairs $(T_i,{\mathbf{\theta}}_i), i=1,2$ of trees and model parameters, we define the distance $d((T_1,{\mathbf{\theta}}_1), (T_2,{\mathbf{\theta}}_2))$ to be the distance between the induced probability distributions on characters $\Omega^n$. Since the GTR model is identifiable (Allman et al. 2008), the map from pairs $(T,{\mathbf{\theta}})$ to probability distributions on $\Omega^n$ is injective, that is, one to one, so that distinct trees always induce distinct distributions. This is also the case for the two-state symmetric model and GTR model with across-site Gamma rate heterogeneity. It follows that the Hellinger and JS metrics on distributions induce metrics on pairs $(T,{\mathbf{\theta}})$.

The total variation metric between distributions is defined by $d_{TV}(p,q)=\sum_{\mathbf{s}}|p(s)-q(s)|$. We did not explore properties of this metric when preparing this paper, but methods to compute the total variation metric are included in the software.

### Sample Size Calculation

When estimating the Hellinger, Kullback–Leibler, and JS distances, it is helpful to determine the smallest size sample needed to be reliably within a given tolerance of the true distance. The estimate

\begin{equation*}
R_{m}=1-\frac{1}{2m}\sum_{i=1}^{m}\left(\sqrt{\frac{q({\mathbf{s}}_{p,i})}{p({\mathbf{s}}_{p,i})}}+\sqrt{\frac{p({\mathbf{s}}_{q,i})}{q({\mathbf{s}}_{q,i})}}\right)
\end{equation*}

is unbiased for the squared Hellinger distance $\mu_0=d_H(p;q)^2$. Also, for large $m$, $R_m$ is approximately normally distributed with variance $\sigma_0^2/m$, where $\sigma_0^2$ is the variance of $R_{m=1}$. We can obtain provisional estimates $\mu_0$ and $\sigma_0^2$ from a pilot run of size $m_0$. Each of the $m_0$ realizations produces an estimate of $R_1$ and so their mean and variance are unbiased estimates for $\mu_0$ and $\sigma_0^2$. Absolute or relative error are standard criteria for determining an appropriate sample size $m$ in this situation. For example, to require the estimate $R_m$ to have an absolute error of $\tau$ with probability $1-\beta$ requires

(2) \begin{equation*}\label{eq:02} m \geq \frac{z_{\beta/2}^{2} \sigma_{0}^{2}}{\tau^2(2\sqrt{\mu_0}-\tau)^2}, \end{equation*}

where $z_{\beta}$ is the upper $\beta$ point of the standard normal distribution (e.g., $z_{0.025}=1.96$). If instead we require a relative error of $\alpha$, this is equivalent to using absolute error $\tau=\alpha\sqrt{\mu_0}$. Estimated values of $\mu_0$ and $\sigma_0^2$ from the pilot run are used in (2) to estimate $m$.

The estimators described above can be improved using the control variate method (Morgan 1984, Chapter 7, p. 171) to give unbiased estimators which achieve the same precision with fewer samples that is smaller values of $m$. We have implemented these improved estimators in the software and more information is given in the Supplementary appendix available on Dryad. We found the reduction in $m$ varied very substantially with each data set.

**Figure 1.**
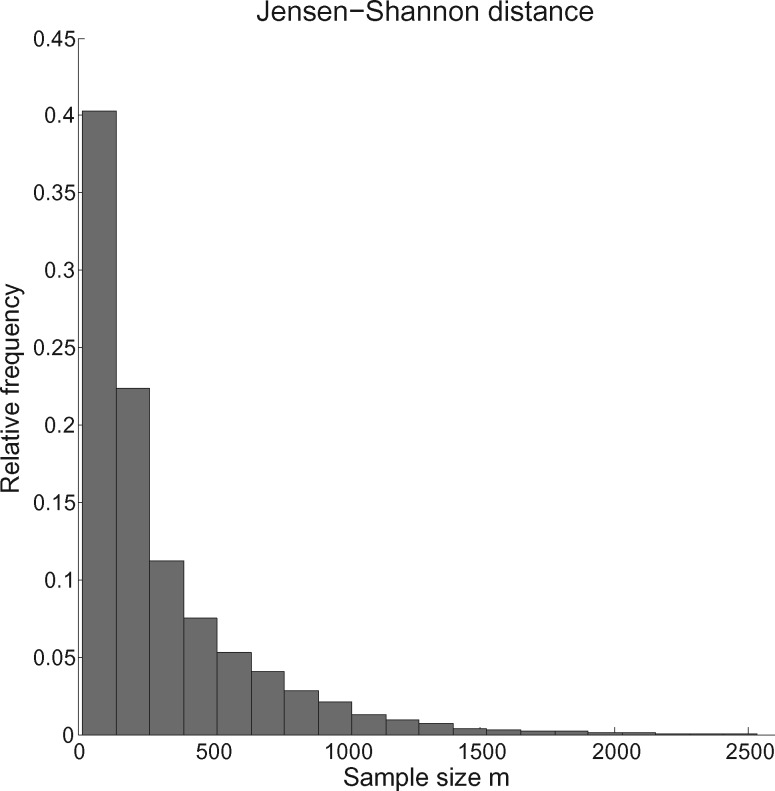
A histogram of estimated values of $m$ for the comparison of every pair of gene trees in the data set of 106 gene trees due to Rokas et al. 2003, using the two-state symmetric model and JS distance. It can be seen that the distance between most pairs of trees in the data set can be estimated accurately with fewer than 2000 samples. In terms of computational cost, this is similar to the cost of computing the likelihood for an alignment of length 2000 for each pair of trees.

In order to explore the possible values for $m$ that might be required for experimental data sets, we estimated $m$ for the JS distance between every pair of gene trees contained in the data set of 106 yeast gene trees on 8 yeast species (Rokas et al. 2003). Figure 1 shows a histogram of estimated values for $m$. Estimation was performed in order to achieve a relative error of $\alpha=5\%$ with probability $1-\beta=80\%$. The figure shows that fairly accurate distances can be obtained using reasonably small sample sizes.

### Missing Taxa

Suppose $T_A$ and $T_B$ are trees with taxon sets $A$ and $B$. Let $p$ and $q$ denote probability mass functions on characters induced by $T_A$ and $T_B$, respectively. Very commonly $A \neq B$ and $A \cap B \neq \emptyset$ but many tree-metrics cannot be computed under these assumptions. We consider two approaches for computing distances when taxon sets differ between trees.

#### Common taxa method

The analysis is restricted to $A\cap B$ by cropping the trees down to the common taxon set. The resulting reduced trees can then used to obtain the distance measures considered in this article.

#### Augmentation method

This method can only be used for our probabilistic distance measures and does not apply to the BHV metric. The strategy here is that we extend the definition of $p$ from $\Omega^{|A|}$ to $\Omega^{|A\cup B|}$ in such a way that the extended distribution is uniform on $\Omega^{|B\setminus A|}$. This is done as follows. Any element ${\mathbf{s}}\in \Omega^{|A\cup B|}$ can be decomposed into three parts corresponding to the taxa in each of the sets $A\setminus B$, $A\cap B$, and $B\setminus A$ and these parts are denoted ${\mathbf{s}}_{A\setminus B}$, ${\mathbf{s}}_{A\cap B}$, and ${\mathbf{s}}_{B\setminus A}$, respectively. Since there are $|\Omega|^{|B\setminus A|}$ possibilities for ${\mathbf{s}}_{B\setminus A}$, the uniform assumption implies that the probability of each possibility is $|\Omega|^{-|B\setminus A|}$. If the extension of $p$ to $\Omega^{|A\cup B|}$ is denoted $p_{A\cup B}$, then we define

\begin{equation*}
p_{A \cup B}({\mathbf{s}}) = p_{A \cup B}({\mathbf{s}}_{A \setminus B},{\mathbf{s}}_{A \cap B},{\mathbf{s}}_{B
 \setminus A})=\frac{p({\mathbf{s}}_{A \setminus B},{\mathbf{s}}_{A \cap
 B})}{|\Omega|^{|B\setminus A|}}
\end{equation*}

for all ${\mathbf{s}}\in \Omega^{|A\cup B|}$ where $p({\mathbf{s}}_{A \setminus B},{\mathbf{s}}_{A \cap B})$ denotes the original distribution on $\Omega^{|A|}$. Probabilistic distances between $T_A$ and $T_B$ can be computed by extending $p$ and $q$ to $A\cup B$ and basing the distance on these extended distributions. The uniform distribution is used as it represents a condition of maximal uncertainty of the position on the trees of the missing taxa.

## Results

We now look at properties of the probabilistic distance measures in several scenarios. The aim is to illustrate possible advantages and disadvantages in comparison to existing metrics, especially the BHV metric.

### Scaling Edges

**Figure 2.**
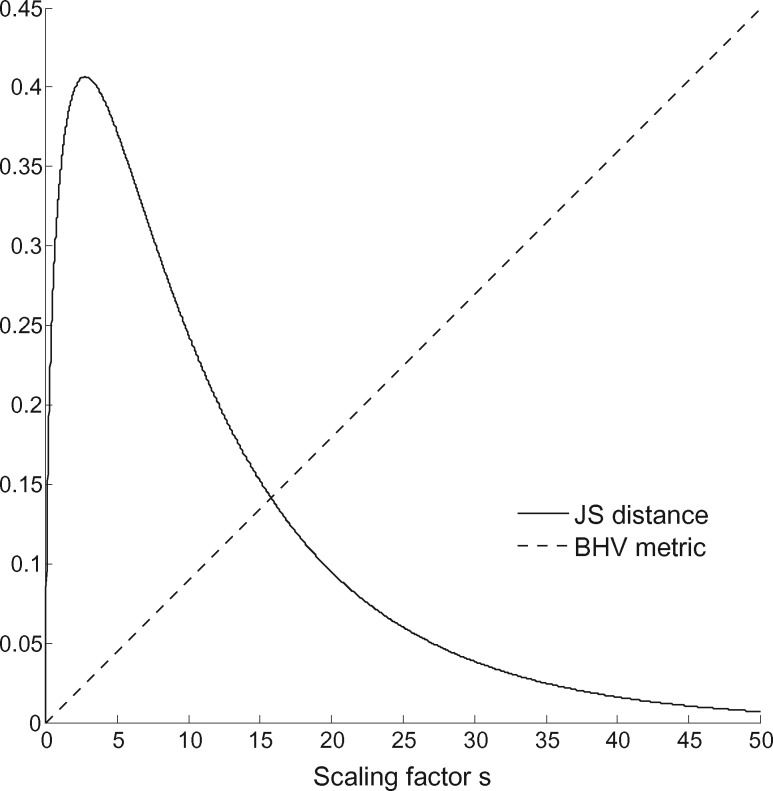
JS distance and BHV metric between two random 16-taxon trees $T_1$ and $T_2$ with branch lengths scaled by a factor $s$.

We consider two trees $T_1=(\tau_1,\mathbf{\ell}_1)$ and $T_2=(\tau_2, \mathbf{\ell}_2)$ on a shared set of $n$ taxa, where $\tau_i$ and $\mathbf{\ell}_i$ are the topology and vector of edge lengths on the trees respectively. Here the two topologies were sampled from a Yule distribution and the edge lengths were sampled from a Gamma distribution with mean 0.1 and variance 0.005. The edge lengths on both trees were then scaled by a factor $s$ and the quantity 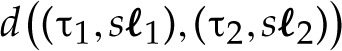 was computed for different values of $s$ using the probabilistic distances and the BHV metric: here $s\mathbf{\ell}_i$ is the vector of edge lengths $\mathbf{\ell}_i$ multiplied by $s$. Distances were calculated using the two-state symmetric substitution model. Figure 2 shows how the Jensen–Shannon (JS) metric does not behave like the BHV metric under this scaling. Note that the absolute values of JS and BHV distances cannot be compared directly (in fact the BHV axis has been rescaled). As we make the edge lengths on both trees increasingly long, the BHV metric increases linearly. However, under the same limit, the distribution of sequence data at each leaf becomes independent of the distribution at any other leaf, and so (due to saturation) both trees give rise to the same distribution on characters under this limit. Therefore, the probabilistic distance between the trees reduces to zero as $s$ increases whereas BHV increases linearly. For values of $s$ which are more meaningful from a biological perspective (e.g., $s$ between zero and five) the JS metric also depends non-linearly on $s$. Hellinger distance and Kullback–Leibler divergence behave similarly to JS distance. This clearly demonstrates the fundamental difference between probabilistic distances and existing distances which use edge-length information.

**Figure 3.**
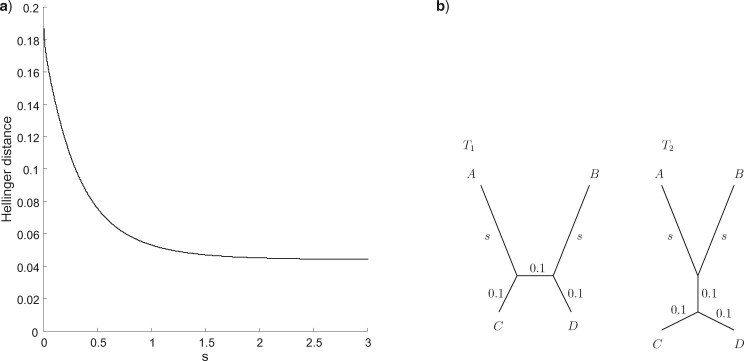
a) Hellinger distance between the two trees shown in b), as a function of $s$, the length of the long pendant edges. The BHV distance does not vary with $s$.

Probabilistic distances behave differently from existing metrics when the trees contain long edges. This is particularly relevant to situations when trees might be subject to long branch attraction artefacts. To illustrate this, we consider trees in the so-called Felsenstein zone and Faris zone (Felsenstein 2008). The trees are shown in Figure 3b. Each represents an alternative hypothesis in the presence of two long edges. As the edge length $s$ increases, the probabilistic distances between the trees decrease to a constant, as shown in Figure 3a. In contrast, the BHV distance does not vary with $s$. The probabilistic distances correctly capture the fact that distinguishing one tree from the other as $s$ increases is difficult since they induce similar distributions on sequence data.

### Missing Taxa

**Figure 4.**
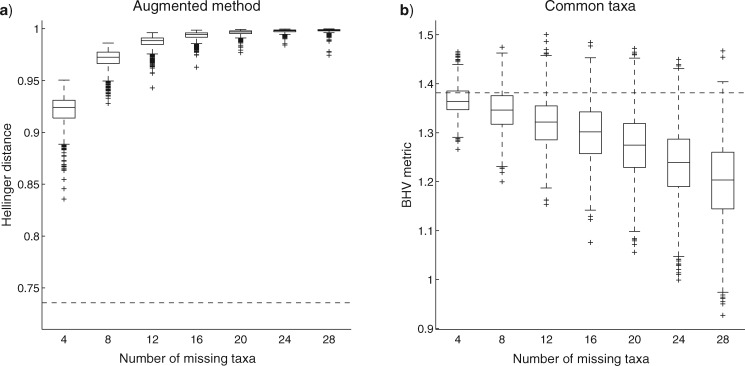
Sampling distribution of distance between two trees for different levels of random deletions of taxa using a) augmented method with Hellinger distance, and b) common taxa method with the BHV metric. The initial pair of trees have the same 100 taxa with the same topology but different (random) edge lengths. The dashed horizontal line is the distance/metric between the initial pair of trees (before any deletions).

**Figure 5.**
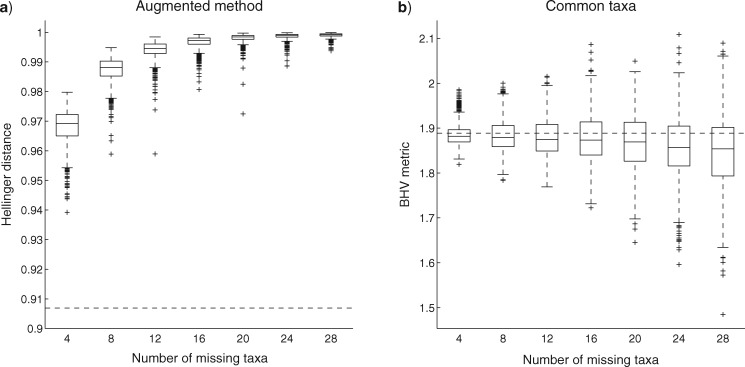
Sampling distribution of distance between two trees for different levels of random deletions of taxa using a) augmented method with Hellinger distance, and b) common taxa method with the BHV metric. Both trees have 100 taxa with different (random) edge lengths, with one tree generated at random and the other tree determined using 10 subsequent SPR operations. The dashed horizontal line is the distance/metric between the initial pair of trees (before any deletions).

We investigate the effect of missing taxa on tree distances by first considering a tree with 100 taxa, again with topology sampled from a Yule distribution. Two copies of this topology were made and then trees constructed by assigning edge lengths independently to each topology from a Gamma distribution with mean 0.1 and variance 0.005. Each tree was then subjected to random deletions of the same number of taxa. By repeating the random deletion many times on the fixed pair of trees, and computing the distance each time, we obtain a distribution of distances between the two trees for a given proportion of deletion on each tree; see Figure 4. We also looked at the effect of random deletions of the same number of taxa from two trees with different topologies: the first being another tree with 100 taxa and the second being determined by applying 10 random subtree prune and regraft (SPR) operations to the first; see Figure 5. In both figures, we compare the augmentation method using Hellinger distance with the common taxa method using the BHV metric. The augmentation method cannot be applied to the BHV metric, since the method is intrinsically probabilistic.

The figures show that as the number of missing taxa increases, the Hellinger distance increases towards its upper bound of 1 and the BHV metric decreases. The decrease in the BHV metric is more substantial in Figure 4 where both initial trees have the same topology (before deletions). These trends were observed in several replicate experiments and in different sizes of trees; results are given in the Supplementary Appendix available on Dryad. Overall these figures show a desirable property of using the augmentation method over the common taxa method, namely that distances between trees increase as we have more uncertainty about the trees due to missing taxa. The probabilistic distance measures with common taxa method behave similarly to the BHV metric with the same method: results obtained with the Hellinger distance and common taxa method are given in the Supplementary Material available on Dryad.

### Incorporating Substitution Model Parameters

**Figure 6.**
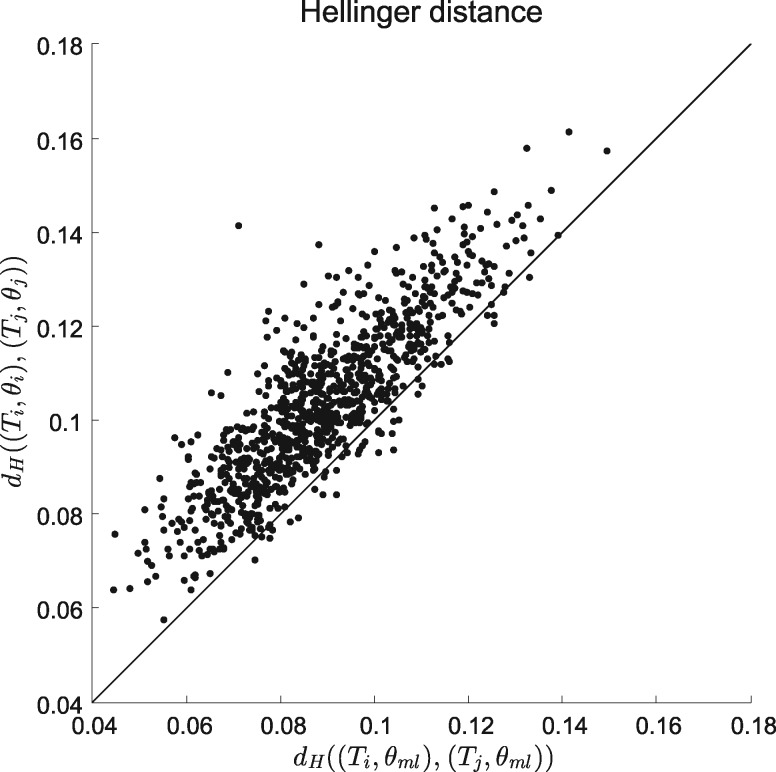
Comparison of Hellinger distance between pairs of trees $T_i$ and $T_j$ using overall ML substitution parameters ${\mathbf{\theta}}_{ML}$ with that using individual ML substitution parameter ${\mathbf{\theta}}_i$. The trees used are ML trees $T_1,\ldots,T_{100}$ obtained from 100 bootstrap replicates of the primate data set.

We now investigate the distribution of distances calculated over biologically plausible trees and their substitution parameters for an experimental data set of primate DNA data (Huelsenbeck and Ronquist 2001). We analyzed the data set using the PHYML program (Guindon and Gascuel 2003) assuming the GTR model with Gamma rate heterogeneity. This gave us the maximum likelihood (ML) tree and its model parameters ${\mathbf{\theta}}_{ML}$ together with a set of 100 bootstrap replicates of trees $T_i$, each with their (ML) model parameters ${\mathbf{\theta}}_i$. For each pair of trees in the bootstrap sample, we calculated the Hellinger distance between trees using the same (ML) model parameters, $d_H((T_i,{\mathbf{\theta}}_{ML}),(T_j,{\mathbf{\theta}}_{ML}))$ and between trees using the tree-parameter pairs, $d_H((T_i,{\mathbf{\theta}}_i),(T_j,{\mathbf{\theta}}_j))$. Pairwise plots of these measures are given in Figure 6. It is clear that the distances are nearly always increased when taking proper account of the substitution parameter values. Similar results were obtained for Kullback–Leibler divergence and Jensen–Shannon distance.

### Phylogenetic Islands

The term *phylogenetic island* has been used to refer to modes in multimodal posterior distributions, especially when these modes correspond to distinct tree topologies. In this section, we study two data sets for which posterior samples have previously been found to contain distinct clusters of trees when the samples are analyzed with metrics based on topological differences between trees. We compute probabilistic distances between trees in posterior samples and perform multidimensional scaling (MDS) using these distances (Hillis et al. 2005). This leads to contrasting probabilistic interpretations of the results for the two data sets.

**Figure 7.**
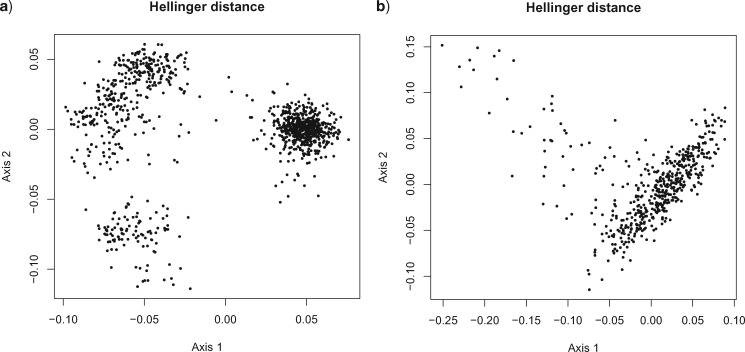
MDS of the pairwise Hellinger distance between a) posterior sample of 1000 trees from the tetrapod data set under GTR+$\Gamma$ model and b) posterior sample of 500 trees from dengue fever data set under GTR+$\Gamma$+I substitution model with uncorrelated lognormal-distributed relaxed molecular clock.

The first data set consists of 1949 nucleotides from 27 tetrapod species (Hedges et al. 1990). The alignment was analyzed in MrBayes (Huelsenbeck and Ronquist 2001) using the GTR model with Gamma rate heterogeneity. The analysis used a burn-in of 1 million iterations followed by another 1 million iterations, sampled every 1000 iterations, in order to obtain a posterior sample of 1000 trees $T_i$ and their associated model parameters ${\mathbf{\theta}}_i$, $i=1,\ldots,1000$. The Hellinger distance was estimated for each pair $(T_i,{\mathbf{\theta}}_i), (T_j,{\mathbf{\theta}}_j)$, $i\neq j$, and these distances were analyzed with MDS. The results are shown in Figure 7a. The second data set consisted of 1485 nucleotides from 17 dengue virus serotype 4 sequences (Drummond and Rambaut 2007). This alignment was analyzed using a GTR model with Gamma rate heterogeneity and invariant sites using an uncorrelated lognormal distributed relaxed molecular clock. The BEAST software was used to perform the analysis (Drummond and Rambaut 2007), using an xml file provided with the software, and 500 pairs $(T_i,{\mathbf{\theta}}_i)$ were sampled from the posterior. Figure 7b shows the results of applying MDS to the Hellinger distances between these pairs.

Previous analyses of these data sets have revealed distinct clusters in posterior samples when distances are measured using topological information alone. Whidden et al. (2015) found clusters in the tetrapod trees using the SPR metric. For the dengue fever sequences, Kendall and Colijn (2015) considered a family of metrics parametrized by $\lambda\in [0,1]$. In the case $\lambda=0$ the metric retains only topological information, and using this metric to perform MDS reveals several distinct clusters in the posterior sample, described by Colijn and Kendall as phylogenetic islands. A figure showing MDS with the Kendall–Colijn metric is contained in the Supplementary Material available on Dryad. MDS with the unweighted Robinson-Foulds metric gives similar results for this data set.

The MDS results obtained using the Hellinger distance differ for the two data sets. For the tetrapod data set, the MDS plot shows distinct clusters of trees. The three clusters correspond to distinct topological regions in tree-space. On the other hand, MDS for the probabilistic distances between dengue fever trees did not reveal any clusters in the posterior sample, as shown in Figure 7b. The clusters obtained with the Kendall–Colijn metric do not correspond to separate regions in this plot (see Supplementary Material available on Dryad). As seen in previous examples in this article, two trees with different topologies can induce similar distributions on sequence data for particular choices of edge lengths and substitution model parameters. The same phenomenon is at play for the dengue fever trees: although the posterior sample contains distinct clusters of topologies, trees in different clusters are in fact giving rise to similar distributions of nucleotides. The interpretation of the results is therefore different in the two cases. First, for the tetrapod data, it appears that a single tree together with the GTR model and Gamma rate heterogeneity is not able to explain the information in the sequence alignment. One possibility is that the substitution model is misspecified and a more sophisticated model is required; a second is that the data have arisen from a nontree-like process, such as a mixture of trees. Secondly, for the dengue fever data, it appears that several distinct groupings of topologies are consistent with the data, but regarding the trees as probability models, these groupings lack meaning as trees in different clusters represent similar distributions on characters. If more sequence data were available, and under the assumption that these sequences were generated by the same evolutionary process, we would expect the single cluster in Figure 7b to become tighter, and correspondingly, for the variability in topology in the posterior sample to be reduced.

### Computing times

The time taken to estimate distances depends on the sample size and hence on the degree of accuracy required by the user. The time taken to compute all $5565$ distances for the yeast data in Figure 1 was $3$ min. Similarly, the time taken to compute all $4950$ distances between trees in the primate bootstrap sample using the GTR model with Gamma rate heterogeneity was 151 minutes. In both cases, the sample size was estimated to achieve a relative error of $\alpha=5\%$ with probability $1-\beta = 80\%$. Calculations were performed using a desktop computer with an Intel(R) Core i7-4790S processor running at 3.20 HGz.

## Conclusions

We have provided methods for computing probabilistic distances between phylogenetic trees based on simulation that give qualitatively different result from the BHV metric and other metrics. Unlike other methods that are purely based on topology and/or edge lengths on the trees, these methods are based on the underlying probability distribution on genetic sequence data induced by the trees. The methods have been extended to deal with trees that do not share the same set of taxa. We envisage probabilistic distances being used as an alternative to existing metrics in any postprocessing of phylogenies which involves a metric.

## Availability

Open source java software is available from www.mas.ncl.ac.uk/~ntmwn/probdist and the source code is also included in the online supplementary material. The software runs under Mac OSX, Gnu/Linux, and Windows.

## Supplementary Material

Data available from the Dryad Digital Respository: http://dx.doi.org/10.5061/dryad.69bb2.

## Funding

This work was supported by a Ph.D studentship to M.G. funded by Newcastle University.
