# Corrigendum to: “Probabilistic Distances Between Trees”

**DOI:** 10.1093/sysbio/syx083

**Published:** 2017-11-22

Garba M.K., Nye T.M.W., Boys R.J. 2017. Probabilistic Distances Between Trees. Sys. Biol. DOI: 10.1093/sysbio/syx080.

The following sentence was incorrect:

“The total variation metric between distributions is defined by $dTV(p,q) = s \in_n |p(s)-q(s)|$. This could be used to defined a distance between trees, but since it cannot be expressed as an expectation, sampling methods cannot be used to compute the metric approximately. As a result, we have not implemented methods for computing *dTV*.”

The correct sentence is below:

“The total variation metric between distributions is defined by $d_TV (p,q) = \sum_s |p(s)-q(s)|$. We did not explore properties of this metric when preparing this paper, but methods to compute the total variation metric are included in the software.”

This has now been corrected in the original article.

